# The City Infant Faces Database: A validated set of infant facial expressions

**DOI:** 10.3758/s13428-017-0859-9

**Published:** 2017-02-15

**Authors:** Rebecca Webb, Susan Ayers, Ansgar Endress

**Affiliations:** 1 Centre for Maternal and Child Health Research, City University London, London, EC1V 0HB UK; 2 Department of Psychology, City University London, London, UK

**Keywords:** Infant faces, Emotional expression, Face database, Stimuli

## Abstract

Adults need to be able to process infants’ emotional expressions accurately to respond appropriately and care for infants. However, research on processing of the emotional expressions of infant faces is hampered by the lack of validated stimuli. Although many sets of photographs of adult faces are available to researchers, there are no corresponding sets of photographs of infant faces. We therefore developed and validated a database of infant faces, which is available via e-mail request. Parents were recruited via social media and asked to send photographs of their infant (0–12 months of age) showing positive, negative, and neutral facial expressions. A total of 195 infant faces were obtained and validated. To validate the images, student midwives and nurses (*n* = 53) and members of the general public (*n* = 18) rated each image with respect to its facial expression, intensity of expression, clarity of expression, genuineness of expression, and valence. On the basis of these ratings, a total of 154 images with rating agreements of at least 75% were included in the final database. These comprise 60 photographs of positive infant faces, 54 photographs of negative infant faces, and 40 photographs of neutral infant faces. The images have high criterion validity and good test–retest reliability. This database is therefore a useful and valid tool for researchers.

**Electronic supplementary material:**

The online version of this article (doi:10.3758/s13428-017-0859-9) contains supplementary material, which is available to authorized users.

The survival of human infants depends on appropriate care by adults (Bjorklund, 1997). Therefore, successful early relationships between infants and their primary caregivers are critical to ensuring the appropriate development, and even the survival, of an infant (Lorenz, 1943). Lorenz argued that the stereotypical features of an infant (large foreheads, large eyes, close-set features positioned low on the face) trigger an innate response in human adults, which encourages care-taking behavior, affective orientation toward the infant, and decreased aggression (Lorenz, 1943), a proposal that has received considerable empirical support. For example, Alley (1981) manipulated the head shapes of infants and asked participants to rate the images on perceived cuteness. The results showed that as the head shape changed as it would with aging, perceived cuteness decreased. In a functional magnetic resonance imaging study, participants were asked to view images of infant faces in which the faces had been manipulated to have either low or high baby schema. These results showed that baby schema activated the nucleus accumbens, a brain structure that has been found to mediate reward processing and appetitive motivation (Glocker, Langleben, Ruparel, Loughead, Valdez, et al., 2009). Additionally, undergraduate students who were asked to rate the cuteness of infants and their desire to look after the infant were more likely to want to look after an infant they had rated as being cute (Glocker, Langleben, Ruparel, Loughead, Gur, & Sachser, 2009). Furthermore, it has been found that, when adults perform tasks that require focused attention, they do such tasks more carefully after looking at images of babies, suggesting that viewing images of infants can increase careful behavior (Nittono, Fukushima, Yano, & Moriya, 2012). This research has shown the importance of adult processing of infant faces to enabling the survival of the species.

The perception of cuteness orients adults’ attention toward infants, but it does not inform them about an infant’s current needs. As a result, parents must also be able perceive their infant’s emotional cues successfully to ensure their infants psychological needs are met. Indeed, parents who are attuned to their infant’s behavioral and emotional cues are more likely to have securely attached infants than parents who are less sensitive (Ainsworth, 1973). Furthermore, females (who are usually the primary caregiver of an infant across primates and in humans; Marlowe, 2000) are more accurate at identifying infant emotional states (Proverbio, Matarazzo, Brignone, Zotto, & Zani, 2007) and mothers are more distracted by infant faces than nonmothers (Thompson-Booth et al., 2014). Further, evolutionary theories posit that the development of infant facial expressions has been designed by natural selection to communicate important information to the caretaker about the emotional state of the infant, arguably increasing the infants chance of survival (Babchuk, Hames, & Thompson, 1985). This shows the importance of being able to accurately identify facial expressions to develop a strong parent–infant bond and secure attachment, and therein perhaps to increase the chances of infant survival.

The perception of infant emotional expressions is therefore an important research area that might provide important insights into infant facial communication and its influence on others. For example, biased or accurate perception of infant emotion might predict a number of outcomes, such as parent-infant interaction, parental mental health or infant perception of peer emotion. However, currently only one set of infant images is available to researchers (Pearson, Cooper, Penton-Voak, Lightman, & Evans, 2010; on request from the authors). This image set has been previously validated on 29 students with high agreement ratings for the images (between 95% and 100%). This image set is restricted to 30 images and does not include images of the same infant showing different emotions. Developing a database with more images to choose from can aid research into the relationship between perception of infant emotion and mother infant interaction, maternal mental health and infant perception of other-infant emotion. Additionally, the availability of different emotions from the same infant reduces other types of variability such as differences in infant attractiveness that would arise when emotions are shown on faces of different infants. Furthermore, having a normed set of infant faces provides researchers with a valuable tool that has the potential to improve aspects of research. For example, these images will facilitate replication across studies and reduce error variance. Therefore, the aim of this study was to develop and validate a standardized database of infant faces that is freely available to authors by e-mailing cityinfantfacedatabase@gmail.com.

## Method

The baby faces database was developed and validated in three stages.

### Stage 1: Development of the database

#### Collection of stimuli

Parents were approached via various social media sites such as Facebook and were asked whether they would be willing to help with research investigating the perception of infant emotion. If parents showed interest and had an infant under the age of 12 months, they were sent an information sheet and consent form. Parents were asked to respond with a minimum of three photographs of their infant showing at least one positive, one negative, and a neutral emotion, as well as their completed consent form. Parents were asked to take the photographs all at the same time of the day and to try to have their baby’s head at the same angle for each photograph. A total of 68 parents consented, and 195 photographs were received.

#### Image processing

All images were edited in Photoshop 2014. The original backgrounds were removed and replaced with a blank background (white for color images and black for black-and-white images). The images were resized to 800 × 1,100 pixels and were saved as color versions. Then the images were converted to black and white and saved again. The color versions are available; however, these have not been fully validated.

**Fig. 1:**
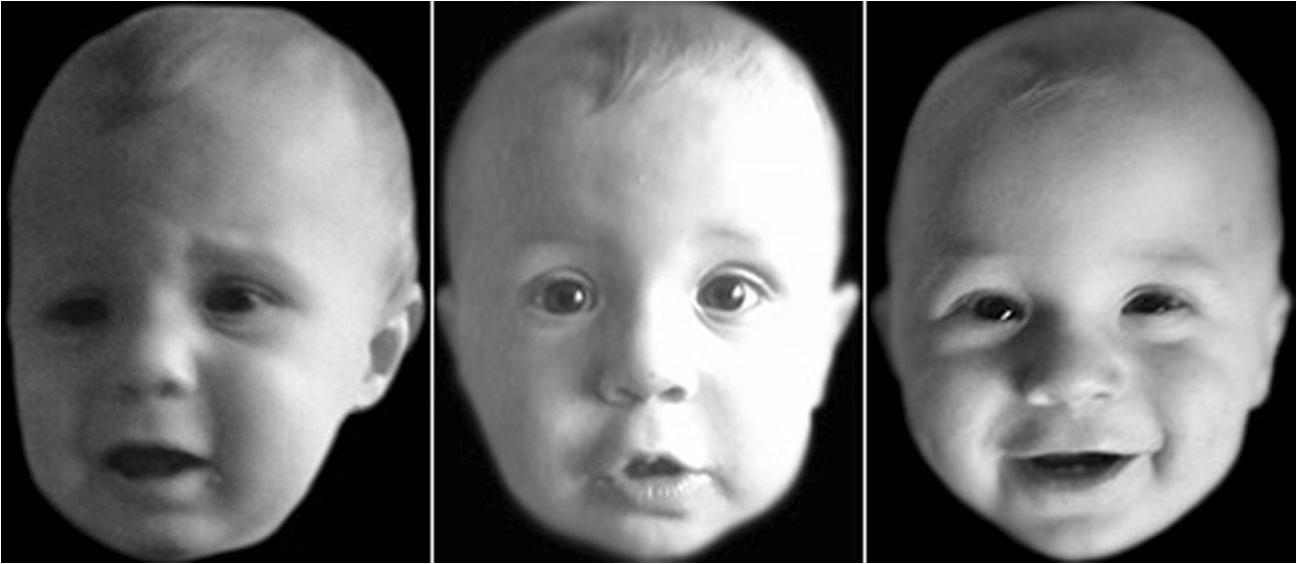
Examples from the City Infant Faces Database. Examples show (from left to right) negative, neutral, and positive expressions

Sample black-and-white images from the database can be seen in Fig. 1.

### Stage 2: Validation of the database

#### Participants

A total of 71 participants took part in the validation of the database. The participants consisted of 41 midwifery and 12 neonatal nursing students from City University London, as well as 18 members of the general public. These were six males, 63 females, and two of undisclosed gender, with a mean age of 28 years (*SD* = 8.52).

#### Measures

##### Ratings of images

The images were rated on six dimensions adapted from (Langner et al., 2010), as follows:

- *Expression*—that is, the expression the participants felt the face was portraying (negative, neutral, positive);
- *Intensity*—that is, how strongly the participants felt the face was showing this emotion (1 *weak*; 5 *strong*);
- *Clarity*—that is, how clear this expression was (1 *unclear*; 5 *clear*);
- *Genuineness*—that is, how genuine the participant felt the expression was (1 *fake*; 5 *genuine*);
- *Affective response*—that is, what emotion the participant felt while viewing the image (negative, neutral, positive);
- *Strength of affective response*—that is, how strongly the participant felt this emotion (1 *weak*; 5 *strong*).

##### Criterion validity

Validity was measured by asking participants to rate the faces from the Pearson image set. This set consists of ten positive facial expressions, ten negative facial expressions, and ten neutral facial expressions. In this dataset validation, the mean accuracies for the images were 99% for positive images, 95% for neutral images, and 100% for negative images (Pearson et al., 2010).

##### Agreement ratings

The images were categorized as either positive, negative, or neutral on the basis of their average rating. The numbers of images that were rated differently for each emotional category were summed, and percent changes between the groups were calculated.

#### Coding data

Since two of the rating scales produced categorical data (Expression and Affective Response), the researcher assigned a number to each emotional category for analysis in IBM SPSS (Version 22) and R (Version 3.3.1): *negative expression/affective response* was coded as 1, *neutral expression/affective response* was coded as 2, and *positive expression/affective response* was coded as 3.

#### Apparatus

For the students, the images were presented in the classroom using Microsoft Office PowerPoint 2007 and were projected onto an 80-in. projector screen using the projector available. For members of the general public, the study was Internet-based, using the Qualtrics survey software (Qualtrics (2017), Provo, UT).

#### Procedure

A total of 255 images were tested: 195 black-and-white images from the database, as described above; 30 of the same images, selected at random, presented in color, to test for any differences between the black-and-white and color images in the perception of emotional expression; and the 30 images from the Pearson image set.

These images were rated by the midwifery and neonatal nursing students. To ensure that all the images were rated, and to minimize the burden on participants, different participants rated different images. This was done in a series of group sessions, and the images were randomly selected for each group. Each image was rated by at least 26 participants. Information on the average validation scores for each individual image is available in online supplemental materials.

All images for the midwifery and neonatal nursing students were presented using Microsoft Office PowerPoint 2007 and projected onto an 80-in. projector screen in the classroom. Each image measured (height × width) 14 cm × 10.6 cm in Microsoft Office PowerPoint. Each image was shown for 20 s. In the top left-hand corner was the image number (i.e., Image 1), so participants could match the image up with the answer booklet he or she had been given.

The members of the general public were recruited through a psychology graduate mailing list, a university mailing list, social media sites such as Facebook, and snowball recruitment. All 255 images were randomized and uploaded to the Qualtrics online survey software (Qualtrics, Provo, UT, 2017). As before, the images were presented for 20 s, and then participants rated them. Participants were given two practice trials at the beginning of the testing session; one of these was untimed, so that participants could ask for clarification on the rating scales, if needed, or review the instructions.

#### Criteria for inclusion in the database

The criteria for inclusion were based on the proportions of participants who classified each image as positive, negative, or neutral. Only images with interrater agreement of the emotion of at least 75% were retained in the database, even if this meant that only one or two images were left in the database per infant. (However, in our comparisons to Pearson’s database, we also include images from the latter database that have agreement ratings of less than 75%.) This cutoff was based on the average percentage agreements found in other studies of images of adult emotional expressions (i.e., the percentage of people who correctly identified the emotion of the image), which range from 71.87% to 82% (Ebner, Riediger, & Lindenberger, 2010; Goeleven, De Raedt, Leyman, & Verschuere, 2008; Langner et al., 2010). This meant that 41 images were removed from the database, with rejected images having poorer agreement that ranged from 46.5% to 70%.

After we had removed the images with poor participant agreement, the percentages of participant agreement for each image were averaged across all images and across each emotional category.

## Stage 3: Test–retest reliability

Test–retest reliability was measured four weeks later with the midwifery students. Participants were invited to take part in the retest study, in which they viewed a subset of 25 of the images they had originally viewed. A total of 41 midwifery students completed the ratings at Time 1, and 19 completed the ratings at Time 2.

### Analysis

To analyze the database, we asked whether a number of factors affected the ratings of the images. These factors were Rater Gender, Infant Gender, Color of the Image (color vs. black/white), and Age of the Infant (infants below 7 months old vs. infants of 7 months and above). We asked these questions for all rating scales—that is, expression, intensity, clarity, genuineness, internal emotion, and strength of internal emotion.

Our analysis strategy was as follows. For each factor and rating scale, we conducted a two-way analysis of variance (ANOVA) with the within-subjects factor Image Category (positive vs. negative vs. neutral) and the within- or between-subjects factor under investigation (e.g., Color).

In the analyses below, we do not apply any correction for repeated comparisons. This is because our goal was to flag factors that might potentially affect image quality, rather than to confirm hypotheses. The following variables were used in the ANOVAs, to investigate their impacts on the rating scales: color vs. black-and-white images, female vs. male infants, male vs. female raters, younger vs. older infants, and differences in the group ratings (midwives vs. neonatal nurses vs. general public). Criterion validity was also assessed by comparing the ratings on the City Database with those on the Pearson image set.

For the within-participant analysis, not all participants completed all the cells of the design; for example, in a two-way ANOVA with the factors Image Category and Color, participants needed to complete 2 × 3 = 6 cells. In the analyses below, we exclude those participants who did not complete all cells and note how many participants have been excluded. It should be noted that for the Strength scale, a large proportion of the data were missing. This was due to many participants rating their internal emotion as being neutral. As a result, rating the strength of a neutral emotion was inappropriate, and this field was left blank by participants.

## Results

Overall, the percentage of raters agreeing on the emotion displayed in the images was 91.76%, with negative images in the database having a 94.87% agreement rate, positive images having a 95.73% agreement rate, and neutral images having an 84.7% agreement rate. Descriptive statistics for the different groups can be found in Table 1.

**Table 1:** Descriptive statistics for each group of images by expression

Image Expression	No. Images in Each Group	Percent Agreement
Positive	60	95.73
Neutral	40	84.7
Negative	54	94.87

### Black-and-white and color images

When black-and-white versus color images were used as the dependent variable (DV), we observed a significant interaction between color and expression category, *F*(2, 114) = 4.55, *p* = *.*013, *η*_p_^2^ = .074. Follow-up ANOVAS revealed that the effect of image category was stronger with black-and-white than with color pictures, but in both cases the positive images were rated as being more positive than the neutral images, which were rated as more positive than the negative images. For more details, please see the supplementary results. When clarity was the independent variable (IV), we observed a main effect of color, *F*(1, 57) = 8.26, *p* = *.*007, *η*_p_^2^ = .127, suggesting that the clarity ratings were higher for the black-and-white images (*M* = 3.47, *SD* = 0.69) than for the color images (*M* = 3.33, *SD* = 0.87). We also observed a main effect of image category, reflecting that the negative and the positive images were rated as being clearer than the neutral images (see the supplementary materials); similar (unsurprising) main effects were found in other analyses as well, and will be presented only in the supplementary materials.

### Difference between male and female raters

The analysis of the intensity rating revealed a significant interaction between the category of the image and the gender of the rater of the images, *F*(2, 132) = 3.44, *p* = *.*04, *η*_p_^2^ = .025. Follow-up analysis showed that females rated the negative and positive images as being more intense than the neutral ones, whereas the males did not vary in their mean intensity ratings across image categories (though their means showed numerically the same tendency as those of females). The same pattern was found with the analysis of clarity, with females rating positive and negative images as being clearer than neutral ones. (Numerically, male raters showed the same tendency, but it did not reach significance.)

### Female and male infants

There was a significant effect of gender on the intensity ratings of the images, *F*(1, 70) = 5.55, *p* = *.*02, *η*_p_^2^ = .0735, showing that female infants received significantly more positive ratings (*M* = 3.41, *SD* = 0.70) than did male infants (*M* = 3.33, *SD* = 0.69). The gender of an infant also affected the strength of the affective response when looking at that infant, *F*(1, 44) = 4.07, *p* = *.*05, *η*_p_^2^ = .085, reflecting that female infants triggered stronger emotions (*M* = 2.86, *SD* = 0.94) than did male infants (*M* = 2.73, *SD* = 0.89).

### Younger versus older infants

The analysis of the genuineness ratings yielded a significant main effect of infant age, *F*(1, 70) = 9.87, *p* = .002, *η*_p_^2^ = .124, suggesting that younger infants were rated as being more genuine (*M* = 3.77, *SD* = 0.71) than were older infants (*M* = 3.67, *SD* = 0.72). When affective response was used as the dependent variable, a significant interaction between the category of the image and the age of the infant was found, *F*(2, 138) = 4.21, *p* = *.*017, *η*_p_^2^ = .057. Follow-up analyses showed that younger infants elicited internal emotions closer to those intended (i.e., negative emotions for negative images) for all image categories, as compared to the older infants (see Table 2).

**Table 2:** Descriptive statistics for younger and older infants by expression

Image Category	Younger Infants		Older Infants	
	*M*	*SD*	*M*	*SD*
Negative	1.4	0.31	1.43	0.35
Neutral	2.04	0.29	2.06	0.23
Positive	2.78	0.22	2.72	0.25

### Criterion validity

In the analysis of the expression ratings, we observed a significant effect of source, *F*(1, 58) = 13.33, *p* < .001, *η*_p_^2^ = .187, suggesting that the ratings were somewhat higher (i.e., more positive) for the City database (*M* = 1.97, *SD* = 0.77) than for the Pearson image set (*M* = 1.91, *SD* = 0.77). See Table 3.

**Table 3:** Comparison between the City Baby Face Database and the Pearson image set for positive and negative images, by rating scales

	Negative Images				Neutral Images				Positive Images			
	City Database		Pearson Image Set		City Database		Pearson Image Set		City Database		Pearson Image Set	
	*M*	*SD*	*M*	*SD*	*M*	*SD*	*M*	*SD*	*M*	*SD*	*M*	*SD*
Expression	1.06	0.09	1.05	0.10	1.90	0.15	1.82	0.32	2.94	0.07	2.85	0.24
Intensity	3.74	0.41	4.17	0.60	2.91	0.65	2.87	0.73	3.54	0.43	3.37	0.58
Clarity	3.74	0.48	4.15	0.72	2.92	0.72	2.77	0.81	3.76	0.42	3.48	0.59
Genuineness	3.66	0.64	3.92	0.90	3.73	0.55	3.58	0.69	4.11	0.47	4.02	0.58
Affective response	1.45	0.31	1.38	0.34	2.03	0.23	1.93	0.30	2.72	0.22	2.64	0.33

In terms of expression and internal emotion, 1 = *negative*, 2 = *neutral*, 3 = *positive*.

When intensity was used as the dependent variable, we again observed a significant effect of source, *F*(1, 58) = 4.16, *p* = *.*046, *η*_p_^2^ = .0669, showing that the Pearson images (*M* = 3.47, *SD* = 0.83) were rated as being more intense than the City images (*M* = 3.40, *SD* = 0.62). There was also a significant interaction between source and image category, *F*(2, 116) = 18.94, *p* < .0001, *η*_p_^2^ = .246. Follow-up analyses revealed that, for both databases, negative images were rated as being more intense than positive images, which in turn were rated as more intense than neutral images. However, this effect was somewhat more pronounced for the Pearson image set, especially for negative images. A similar pattern was found when clarity was used as the DV, where the negative images in the Pearson image set were rated as being the clearest among the three categories, whereas negative images did not differ in clarity from positive images in the City database.

For analysis of the genuineness rating, we found an interaction between image category and source, *F*(2, 114) = 12.83, *p* < .00001, *η*_p_^2^ = .184. Follow-up analyses revealed that, for the City database, the positive images were rated as being the most genuine, whereas neutral and negative images did not differ in genuineness. In contrast, for the Pearson image set, the neutral images were rated as being the least genuine, with no difference between the positive and negative images. Our analysis of the ratings of affective response revealed a significant effect of source, *F*(1, 57) = 17.19, *p* < .001, *η*_p_^2^ = .232, suggesting that the ratings were more positive for the City database (*M* = 2.06, *SD* = 0.58) than for the Pearson database (*M* = 1.98, *SD* = 0.61).

### Reliability testing

To measure how much the perception of each picture changed over time, the midwifery students were asked to rate a subset of the pictures on two occasions. An average rating per image for Time 1 and Time 2 was then calculated. Spearman’s correlation coefficient was calculated from these averages. Excellent test–retest reliability was found for the negative (*r* = .954) and neutral (*r* = .965) images, and good test–retest reliability was found for the positive images (*r* = .655).

#### Percent change analysis

To assess how likely participants were to change their minds about the images between Time 1 and Time 2, the number of occasions was counted on which a participant changed her or his mind for the images, and the percentage of changes was calculated relative to the total number of ratings (changed and unchanged). For negative images, participants changed their minds on 1.89% of the ratings; for neutral images, they changed on 18.75%; and for positive images, the ratings changed for 8.76%. The relationship between these variables was significant, *χ* ^2^(2) = 15.88, *p* < .01.

### Participant group ratings

We compared the ratings across the three groups (midwives, neonatal nurses, and general public) on all rating scales. A main effect of image category was found for all analyses, and will not be reported further. The analysis of the expression ratings yielded a main effect of group, *F*(2, 68) = 3.59, *p* = *.*03, *η*_p_^2^ = .095. A post-hoc test (Tukey’s HSD) revealed that the neonatal nurses had higher ratings than either the midwives or the general public, whereas midwives and the general public did not differ significantly. The analyses of the intensity rating revealed a significant interaction between group and image category, *F*(4, 136) = 4.72, *p* = .001, *η*_p_^2^ = .061. Follow-up analyses revealed that, for the midwives and the general public, the negative images were rated as more intense than the positive images, which in turn were rated as more intense than the neutral images. For the neonatal nurses, in contrast, the positive images were rated as the most intense.

The analyses of the clarity rating revealed a significant interaction between group and image category, *F*(2, 68) = 2.28, *p* = *.*11, *η*_p_^2^ = .063. Follow-up analyses revealed that the midwives and the general public rated the positive and negative images as being clearer than the neutral images, with no difference between the positive and negative images. In contrast, the neonatal nurses rated the positive images as being clearer than the neutral images (see Table 4). When genuineness was used as the DV, a main effect of group, *F*(2, 68) = 11.85, *p* < .001, *η*_p_^2^ = .258, was found, showing that neonatal nurses rated all images as being less genuine than did the other groups. An interaction of group and image category, *F*(4, 136) = 4.13, *p* = *.*003, *η*_p_^2^ = .072, was also found. Follow-up analyses showed that the positive images were rated as being more genuine than the neutral or negative images, with no difference between the latter two categories. This effect was most pronounced for midwives.

**Table 4:** Comparison between midwives, neonatal nurses, and the general public for positive, negative, and neutral images, by rating scales

	Negative Images						Neutral Images						Positive Images					
	Midwives		Neonatal Nurses		General Public		Midwives		Neonatal Nurses		General Public		Midwives		Neonatal Nurses		General Public	
Rating Scale	*M*	*SD*	*M*	*SD*	*M*	*SD*	*M*	*SD*	*M*	*SD*	*M*	*SD*	*M*	*SD*	*M*	*SD*	*M*	*SD*
Expression	1.08	0.96	1.08	0.09	1.04	0.05	1.89	0.17	2.00	0.18	1.93	0.09	2.94	0.07	2.99	0.18	2.93	0.08
Intensity	3.78	0.33	3.23	0.73	3.66	0.53	2.87	0.60	2.64	0.68	3.00	0.77	3.60	0.37	3.73	0.56	3.40	0.53
Clarity	3.75	0.42	3.16	0.73	3.63	0.54	2.77	0.73	2.65	0.79	3.25	0.64	3.81	0.36	3.74	0.61	3.63	0.54
Genuineness	3.55	0.60	2.82	0.57	3.90	0.67	3.64	0.53	2.85	0.87	3.93	0.55	4.11	0.43	3.79	0.60	4.13	0.56
Affective response	1.46	0.29	1.29	0.31	1.46	0.37	2.04	0.25	2.18	0.19	2.01	0.17	2.74	0.19	2.96	0.04	2.63	0.32
Strength of affective response	2.92	0.73	2.97	0.61	2.73	0.89	2.40	0.90	2.91	0.89	2.15	0.97	3.32	0.66	3.53	0.48	2.78	0.70

The analysis of the rating of affective response revealed an interaction between image category and group, *F*(4, 136) = 3.69, *p* = *.*007, *η*_p_^2^ = .014. Follow-up tests showed that, whereas the images generally elicited the internal emotions expected from the image category (i.e., negative emotions for negative images), this relationship was strongest for the neonatal nurses. The ratings of the strength of the affective response were marginally lower in the general public than in the other groups.

### Description of the validated database

The database and norming data can be accessed on request by e-mailing cityinfantfacedatabase@gmail.com. The database contains 154 portrait images, with both black-and-white and color versions available (though the color versions have not been fully validated; researchers should take this into account if considering using a mix of the black-and-white and color images). Black-and-white versions of the images are available in two sizes: 150 × 198 pixels or 800 × 1,100 pixels. Color images have not been resized or normalized in terms of their luminosity or hue. In all, 30 of the infants have photographs showing positive, negative, and neutral expressions. In the case of this database, the positive facial expressions are defined as smiling, laughing, or excited; the negative facial expressions are defined as sad, angry, worried, scared, or distressed. There are a total of 60 positive images, 54 negative images, and 40 neutral images to choose from. Images of 35 girls and 33 boys are included in this database, all from 0 to 12 months of age. Sixty-two of these babies are Caucasian, three are Asian, two Arab, and one Indian. Descriptive statistics, including percentages, can be found in Table 5. For more demographic information about the infants included in this database, please see the online supplemental materials.

**Table 5:** Description of the City database

Variable	*N*	Range	Mean	*SD*
Age	69	0–12 months	6.57 months	2.72
	*N*		Percentage	
Gender				
Male	33		48.5%	
Female	35		51.5%	
Ethnicity				
Caucasian	62		91.2%	
Asian	3		4.4%	
Arab	2		2.9%	
Indian	1		1.5%	

## Discussion

This article reports the development and validation of the City Infant Faces database. The results suggest that this database has excellent face validity, with an average agreement rate of 91.76%, which is higher than that reported in validation studies of adult faces (Ebner et al., 2010; Goeleven et al., 2008; Langner et al., 2010). The database is comparable to other image sets of infant faces (Pearson et al., 2010) in terms of agreement ratings, suggesting good criterion validity. Test–retest reliability was also good for all images, although neutral images showed a somewhat higher rate of changes in ratings across time. Additionally, the results showed that neonatal nurses rated the images as being the least genuine and the most positive and as eliciting the internal emotions they expected from the image category, as compared to midwives and the general public. This suggests that the images should be used with caution in groups of individuals exposed to high levels of extreme infant emotion. Furthermore, it is unclear whether there are consistent differences between the black-and-white and color images with regard to the rating scales. The majority of the color and black and white images had no significant differences between their ratings. This is in line with previous research that has found no benefit of color over black-and-white images in terms of the recognition of stimuli (Marx, Hansen-Goos, Thrun, & Einhäuser, 2014). ANOVAs indicated that the black-and-white images were rated as clearer. However, this analysis was only carried out on a small number of color images (*n* = 30); therefore, the results may be different if all of the color images were analyzed. This should be taken into account if researchers are considering using a mixture of the color and black and white images in their research.

With regard to the effect of infant characteristics on ratings, female infants’ emotions were rated as more intense and as eliciting a stronger affective response. Some research has found that female infants smile more than male infants (Cossette, Pomerleau, Malcuit, & Kaczorowski, 1996) and cry for longer than boys in response to hearing another infant cry (Sagi & Hoffman, 1976); therefore, when female infants display emotions, they arguably do so in a more intense way. However, there are many inconsistencies in this literature. For example one study showed that male infants showed more joy and anger than female infants (Weinberg, Tronick, Cohn, & Olson, 1999). Additionally, Geangu, Benga, Stahl, and Striano (2010) found that male infants between 1 and 9 months of age cried for longer and more intensely than did female infants. Therefore, it is not clear why this result was found, and future research should look into this.

Another interesting finding is that younger infants’ expressions were rated as being more genuine and as eliciting the internal emotions expected on the basis of the expression of the image. This is in line with previous research that has revealed that the younger an infant is, the more likely an adult is to rate the infant as cute and likeable, and the more likely the adult is to want to take care of the infant (Luo, Li, & Lee, 2011; Volk, Lukjanczuk, & Quinsey, 2007). Eliciting stronger internal emotions and adults seeing the emotion expressed by the infant as more genuine may help the infant to survive. This is because the only way that an infant can survive is through the care of adults, and evoking positive reactions from adults is likely to increase caregiving behavior by the adult (Lorenz, 1943; Luo et al., 2011).

A few limitations should be taken into account when using this database. One of the main limitations is that the images were not specifically validated on parents. Although some of the participants who took part may have been parents, this was not measured. Furthermore, because only six males contributed to the ratings for this image set, it is unclear whether this database is valid for use with males. This is because research has shown that women’s attentional bias toward infants is stronger and more stable than men’s (Cárdenas, Harris, & Becker, 2013). Furthermore, females are more consistent at choosing cuter infant faces than are males (Lobmaier, Sprengelmeyer, Wiffen, & Perrett, 2010). The results from this database support this showing that females rated the images as more intense and clearer. As a result, caution should be taken if researchers wish to use this database with males.

Another limitation of the database is that the majority of the infants are Caucasian. Although significant efforts were made to try and recruit babies of different ethnicities, this was unfortunately not successful. Furthermore, due to the naturalistic way these images were taken, not all images were taken at the same time. Therefore, although this could be a possible drawback to the database, it is something that could not be overcome when producing such naturalistic images.

The images in this database are arguably more naturalistic than the images from other databases. This is due to the production of the images, which were taken by parents of their infants’ spontaneous facial expressions in naturalistic environments. On the other hand, most adult face databases are produced by recruiting professional models to act out certain emotional expressions that are in line with the Ekman and Friesen Facial Action Coding System (FACS; Ekman & Friesen, 1978). Furthermore, these images are often taken by a professional photographer under controlled conditions (e.g., the same lighting and the same background).

The differences in how the images in different databases were produced may explain the findings from this study in terms of the negative images. It could be suggested that all of the negative images in this set were rated as less intense and less clear because of the selection process and production of the images. For example, the negative images selected by Pearson et al. (2010) were defined as an “infant actively crying” (p. 625), whereas the definition of negative facial expressions in this database was broader. Crying provides a very powerful message to adults about the needs of an infant (Smith, Cowie, & Blades, 2003), and both photographs and tape recordings of infant crying has been found to alter physiological responses in adults (Boukydis & Burgess, 1982). This could, therefore, be the reason behind the lower ratings for the negative images in this database.

Despite this, the naturalistic nature of these images may be more reflective of infant emotion during parent-infant interaction. This is a clear advantage of the database, since before infants are able to communicate verbally, their facial expressions are one of their main methods of communication. For example, infants will smile in response to attention or to a familiar voice (Trevarthen, 1979) and will show distress if their mother’s face suddenly becomes unresponsive (Adamson & Frick, 2003). Having naturalistic facial expressions in the database may enable researchers to learn more about the processing of infant emotions that parents are likely to see on a day-to-day basis, rather than extreme emotions that may not be seen as often. These images may therefore provide researchers with a new way to investigate maternal sensitivity. Thus, despite the limitations of this database, it has many benefits, and therefore can provide a useful tool for researchers to use when researching infant emotion.

## Electronic supplementary material

Below is the link to the electronic supplementary material.

ESM 1 (DOCX 92 kb)

ESM 2 (CSV 20 kb)
