# Supplementary material for: The City Infant Faces Database: A validated set of infant facial expressions
Source: Behav Res Methods. 2017 Feb 15;50(1):151–9. doi: 10.3758/s13428-017-0859-9 (PMC5809537; doi:10.3758/s13428-017-0859-9)
Supplement: Supplementary file 1 — (DOCX 92 kb) [file 13428_2017_859_MOESM1_ESM.docx]

**Supplementary Results**

**S.1. Descriptive statistics**

In Table S1, we provide descriptive statistics for all rating scales. These statistics are based on all participants who rated the images. As a result, the descriptive statistics might differ slightly for those analyses below that were run only on a subsample (e.g., Neonatal Nurses did not rate color images).

*Table S1: Descriptive statistics. The rating scales are as follows. Expression: 1 = negative, 2 = neutral, 3 = positive. Intensity: 1 = not intense, 5 = very intense. Clarity: 1 = not clear, 5 = very clear. Genuineness: 1 = not genuine, 5 = very genuine. Affective response: 1 = negative, 2 = neutral, 3 = positive. Strength of affective response: 1 = weak, 5 = very strong.*

**Strength of**

**Image Expression Intensity Clarity Genuineness Affective resp. affective resp.**

**Data base category *N M SD M SD M SD M SD M SD M SD***

City Negative 54 1.04 .06 3.65 .63 3.68 .61 3.64 .44 1.15 .18 2.75 .40

Neutral 40 1.89 .32 2.67 .40 2.83 .50 3.58 .43 2.19 .48 2.39 .53

Positive 60 2.98 .05 3.61 .60 3.82 .49 4.15 .29 2.96 .06 3.12 .42

Pearson Negative 11 1.03 .07 4.11 .54 4.07 .56 4.01 .39 1.12 .13 3.07 .35

Neutral 10 1.80 .34 2.88 .61 2.81 .44 3.71 .22 1.92 .49 2.36 .55

Positive 10 2.91 .10 3.51 .68 3.65 .66 4.21 .35 2.88 .14 3.01 .41

In Table S2, we also report the correlations across images among the different rating scales. Intensity, clarity, genuineness and strength of affective response are highly correlated, as are Expression and Affective response.

*Table S2: Correlations among the different rating scales.*

**Strength of**

**Intensity Clarity Genuineness Affect. resp. affect. resp.**

**Expression** -.03 .11 .52 .98 .34

**Intensity** .93 .66 -.07 .75

**Clarity** .72 .07 .75

**Genuineness** .46 .71

**Affect. Resp.** .30

**S.2 Color Vs. Black & White Images**

We analyzed the different rating scales using two-way within-subjects ANOVAs with the within-subject factors expression category (negative vs. positive vs. neutral) and color of the image (colored vs. black/white). Only midwives and the general public rated both color and black/white images. We needed to exclude one participant who did not complete the whole design.

S.2.1 DV: Expression

For expression, we observed a main effect of category in that the negative images had the lowest ratings and the positive images had the highest ratings. We also observed a significant interaction between color and expression category, *F*(2,114) = 4.55, *p* = 0.013, η^2^_p_ = 0.074. Follow-up ANOVAS revealed that the effect of image category was stronger with black/white pictures, *F*(2,114) = 4109, *p* < .00001, η^2^_p_ = 0.986, than with color pictures, *F*(2,114) = 1855, *p* < .00001, η^2^_p_ = 0.97. However, as shown in Table S2, all pairwise differences between negative, neutral and positive images were significant for both color pictures and black/white pictures.

*Table S3: Results of (uncorrected) pairwise t-test investigating the effect of image category (negative vs. neutral vs. positive), for the different predictor variables (color: color vs. black/white images; infant gender; rater gender; infant age: up to 6 months vs. above; source: City database vs. Pearson database; group: neonatal nurses vs. midwifes vs. general public) and the different rating scales (expression; intensity of expression; clarity of expression, genuineness of expression; affective response; strength of affective response). The level column indicates whether the analyses were performed on the entire data set or rather on a subset defined by the predictor variable (e.g., black/white images for the predictor Color). The comparison column indicates which pictures have been compared.*

**Confidence interval limits**

**Predictor Scale Level Comparison Difference lower upper *t* df *p* Cohen’s *d***

**Color Expression Overall Neutral-negative 0.84 0.80 0.88 41.6 58 < .0001 5.41**

**Positive-negative 1.86 1.83 1.90 118.1 58 < .0001 15.37**

**Positive-neutral 1.02 0.98 1.06 49.9 58 < .0001 6.50**

**B/W Neutral-negative 0.84 0.79 0.88 36.9 57 < .0001 4.84**

**Positive-negative 1.87 1.84 1.90 117.7 57 < .0001 15.46**

**Positive-neutral 1.03 0.99 1.08 45.5 57 < .0001 5.98**

**Color Neutral-negative 0.91 0.86 0.97 32. 2 57 < .0001 4.23**

**Positive-negative 1.85 1.79 1.92 57.9 57 < .0001 7.60**

**Positive-neutral 0.94 0.88 1.00 30.5 57 < .0001 4.01**

**Intensity Overall Neutral-negative -0.82 -0.99 -0.64 9.16 58 < .0001 1.19**

**Positive-negative -0.21 -0.31 -0.11 4.26 58 < .0001 0.55**

**Positive-neutral 0.61 0.45 0.77 7.59 58 < .0001 0.99**

**Clarity Overall Neutral-negative -0.83 -1.02 -0.638 8.64 58 < .0001 1.12**

Positive-negative 0.02 -0.09 0.13 0.30 58 .076 0.04

**Positive-neutral 0.84 0.67 1.01 9.77 58 < .0001 1.27**

*Table S3 (continued)*

**Confidence interval limits**

**Predictor Scale Level Comparison Difference lower upper *t* df *p* Cohen’s *d***

**Genuineness Overall** Neutral-negative 0.10 -0.06 0.26 1.22 58 .227 0.16

**Positive-negative 0.47 0.32 0.63 6.11 58 < .0001 0.80**

**Positive-neutral 0.38 0.27 0.48 7.04 58 < .0001 0.92**

**Affective Overall Neutral-negative 0.57 0.49 0.65 14.1 58 < .0001 1.84**

**response Positive-negative 1.24 1.11 1.36 19.7 58 < .0001 2.56**

**Positive-neutral 0.67 0.59 0.75 16.8 58 < .0001 2.18**

**Strength Overall** **Neutral-negative -0.46 -0.79 -0.13 2.80 57 .007 0.37**

**of affective response** Positive-negative 0.23 -0.01 0.46 1.91 58 .061 0.25

**Positive-neutral 0.71 0.42 1.0 4.88 58 < .0001 0.64**

**Infant Expression Overall Neutral-negative 0.85 0.81 0.90 40.7 70 < .0001 4.83**

**gender Positive-negative 1.88 1.85 1.90 136.0 70 < .0001 16.14**

**Positive-neutral 1.02 0.98 1.06 50.2 70 < .0001 5.95**

**Intensity Overall Neutral-negative -0.80 -0.96 -0.63 9.8 70 < .0001 1.16**

Positive-negative -0.09 -0.21 0.03 1.43 70 .157 0.17

**Positive-neutral 0.71 0.55 0.87 9.00 70 < .0001 1.07**

*Table S3 (continued)*

**Confidence interval limits**

**Predictor Scale Level Comparison Difference lower upper *t* df *p* Cohen’s *d***

**Clarity Overall Neutral-negative -0.77 -0.95 -0.59 8.59 70 < .0001 1.02**

Positive-negative 0.11 -0.007 0.23 1.87 70 .066 0.22

**Positive-neutral 0.88 0.72 1.05 10.53 70 < .0001 1.25**

**Genuineness Overall** Neutral-negative 0.07 -0.08 0.22 0.89 70 0.375 0.11

**Positive-negative 0.54 0.40 0.68 7.80 70 < .0001 0.93**

**Positive-neutral 0.47 0.35 0.60 7.51 70 < .0001 0.89**

**Affective Overall Neutral-negative 0.62 0.54 0.70 15.4 70 < .0001 1.83**

**response Positive-negative 1.32 1.20 1.43 22.9 70 < .0001 2.72**

**Positive-neutral 0.70 0.62 0.77 19.3 70 < .0001 2.29**

**Strength Overall Neutral-negative 0.62 0.54 0.70 15.4 70 < .0001 1.83**

**of affective response Positive-negative 1.32 1.20 1.43 22.9 70 < .0001 2.72**

**Positive-neutral 0.70 0.62 0.77 19.3 70 < .0001 2.29**

**Rater Expression Overall Neutral-negative 0.85 0.81 0.90 40.7 70 < .0001 4.83**

**gender Positive-negative 1.88 1.85 1.90 136.0 70 < .0001 16.14**

**Positive-neutral 1.02 0.98 1.06 50.2 70 < .0001 5.95**

*Table S3 (continued)*

**Confidence interval limits**

**Predictor Scale Level Comparison Difference lower upper *t* df *p* Cohen’s *d***

**Intensity Overall Neutral-negative -0.80 -0.96 -0.63 9.76 70 < .0001 1.16**

Positive-negative -0.09 -0.21 0.03 1.43 70 0.157 0.17

**Positive-neutral 0.71 0.55 0.87 9.00 70 < .0001 1.07**

**Females Neutral-negative -0.81 -0.97 -0.65 10.26 61 < .0001 1.30**

Positive-negative -0.05 -0.18 0.07 0.85 61 .397 0.11

**Positive-neutral 0.75 0.60 0.91 9.45 61 < .0001 1.20**

**Males** Neutral-negative -0.28 -1.10 0.55 0.87 5 0.43 0.35

Positive-negative -0.13 -0.67 0.41 0.62 5 0.56 0.25

Positive-neutral 0.15 -0.50 0.79 0.59 5 0.58 0.24

**Clarity Overall Neutral-negative -0.77 -0.95 -0.59 8.59 70 < .0001 1.02**

Positive-negative 0.11 -0.01 0.23 1.87 70 0.066 0.22

**Positive-neutral 0.88 0.72 1.05 10.53 70 < .0001 1.25**

**Female Neutral-negative -0.83 -1.02 -0.64 8.76 61 < .0001 1.11**

**Positive-negative 0.15 0.02 0.27 2.31 61 0.024 0.29**

**Positive-neutral 0.98 0.81 1.15 11.31 61 < .0001 1.44**

**Male** Neutral-negative -0.15 -0.96 0.67 0.47 5 0.658 0.19

Positive-negative -0.01 -0.45 0.43 0.06 5 0.954 0.02

Positive-neutral 0.14 -0.48 0.76 0.57 5 0.593 0.23

*Table S3 (continued)*

**Confidence interval limits**

**Predictor Scale Level Comparison Difference lower upper *t* df *p* Cohen’s *d***

**Genuineness Overall** Neutral-negative 0.07 -0.08 0.22 0.89 70 0.375 0.11

**Positive-negative 0.54 0.40 0.68 7.80 70 < .0001 0.93**

**Positive-neutral 0.47 0.35 0.60 7.51 70 < .0001 0.89**

**Affective Overall Neutral-negative 0.62 0.54 0.70 15.4 70 < .0001 1.83**

**response Positive-negative 1.32 1.20 1.43 22.9 70 < .0001 2.72**

**Positive-neutral 0.70 0.62 0.77 19.3 70 < .0001 2.29**

**Strength Overall Neutral-negative -0.45 -0.68 -0.22 4.0 58 0.0002 0.52**

**of affective response** **Positive-negative 0.35 0.22 0.48 5.3 58 < .0001 0.70**

**Positive-neutral 0.80 0.58 1.03 7.1 58 < .0001 0.92**

**Infant Expression Overall Neutral-negative 0.85 0.81 0.90 40.72 70 < .0001 4.83**

**age Positive-negative 1.88 1.85 1.90 136.0 70 < .0001 16.14**

**Positive-neutral 1.02 0.98 1.06 50.2 70 < .0001 5.95**

**Intensity Overall Neutral-negative -0.80 -0.96 -0.63 9.76 70 < .0001 1.16**

Positive-negative -0.09 -0.21 0.03 1.43 70 0.16 0.1**7**

**Positive-neutral 0.71 0.55 0.87 9.00 70 < .0001 1.07**

*Table S3 (continued)*

**Confidence interval limits**

**Predictor Scale Level Comparison Difference lower upper *t* df *p* Cohen’s *d***

**Clarity Overall Neutral-negative -0.77 -0.95 -0.59 8.59 70 < .0001 1.02**

Positive-negative 0.11 -0.01 0.23 1.87 70 0.066 0.22

**Positive-neutral 0.88 0.72 1.05 10.53 70 < .0001 1.25**

**Genuineness Overall** Neutral-negative 0.07 -0.08 0.22 0.89 70 0.375 0.11

**Positive-negative 0.54 0.40 0.68 7.80 70 < .0001 0.93**

**Positive-neutral 0.47 0.35 0.60 7.51 70 < .0001 0.89**

**Affective Overall Neutral-negative 0.62 0.54 0.70 15.42 70 < .0001 1.83**

**response Positive-negative 1.32 1.20 1.43 22.94 70 < .0001 2.72**

**Positive-neutral 0.70 0.62 0.77 19.34 70 < .0001 2.29**

**Young Neutral-negative 0.65 0.56 0.73 14.74 69 < .0001 1.76**

**Positive-negative 1.39 1.28 1.50 25.14 69 < .0001 3.00**

**Positive-neutral 0.74 0.65 0.83 16.76 69 < .0001 2.00**

**Old Neutral-negative 0.63 0.54 0.72 13.90 69 < .0001 1.66**

**Positive-negative 1.29 1.16 1.41 20.98 69 < .0001 2.51**

**Positive-neutral 0.66 0.58 0.73 17.80 69 < .0001 2.13**

*Table S3 (continued)*

**Confidence interval limits**

**Predictor Scale Level Comparison  lower upper *t* df *p* Cohen’s *d***

**Strength Overall Neutral-negative -0.38 -0.66 -0.10 2.74 69 0.008 0.33**

**of affective response Positive-negative 0.33 0.11 0.54 3.05 70 0.003 0.36**

**Positive-neutral 0.73 0.46 1.01 5.37 70 < .0001 0.64**

**Source Expression Overall Neutral-negative 0.83 0.79 0.88 40.17 58 < .0001 5.23**

**Positive-negative 1.86 1.83 1.89 133.56 58 < .0001 17.39**

**Positive-neutral 1.03 0.98 1.07 48.35 58 < .0001 6.29**

**Intensity Overall Neutral-negative -0.92 -1.09 -0.75 10.73 58 < .0001 1.40**

**Positive-negative -0.31 -0.41 -0.21 6.41 58 < .0001 0.83**

**Positive-neutral 0.61 0.45 0.77 7.78 58 < .0001 1.01**

**City Neutral-negative -0.84 -1.02 -0.66 9.29 58 < .0001 1.21**

**Positive-negative -0.21 -0.31 -0.10 3.96 58 0.0002 0.51**

**Positive-neutral 0.63 0.47 0.80 7.64 58 < .0001 1.00**

**Pearson Neutral-negative -1.30 -1.53 -1.08 11.79 58 < .0001 1.53**

**Positive-negative -0.81 -1.01 -0.60 7.90 58 < .0001 1.03**

**Positive-neutral 0.50 0.29 0.71 4.80 58 0.00001 0.63**

*Table S3 (continued)*

**Confidence interval limits**

**Predictor Scale Level Comparison Difference lower upper *t* df *p* Cohen’s *d***

**Clarity Overall Neutral-negative -0.92 -1.12 -0.72 9.35 58 < .0001 1.22**

Positive-negative -0.10 -0.21 0.01 1.88 58 0.064 0.25

**Positive-neutral 0.82 0.64 0.99 9.34 58 < .0001 1.22**

**City Neutral-negative -0.83 -1.03 -0.62 8.22 58 < .0001 1.07**

Positive-negative 0.01 -0.10 0.13 0.25 58 0.801 0.03

**Positive-neutral 0.84 0.66 1.02 9.22 58 < .0001 1.20**

**Pearson Neutral-negative -1.38 -1.62 -1.14 11.60 58 < .0001 1.51**

**Positive-negative -0.67 -0.91 -0.44 5.76 58 < .0001 0.75**

**Positive-neutral 0.71 0.49 0.93 6.53 58 < .0001 0.85**

**Genuineness Overall** Neutral-negative -0.002 -0.16 0.15 0.03 58 0.979 0.003

Positive-negative 0.39 0.24 0.54 5.20 58 < .0001 0.68

Positive-neutral 0.39 0.28 0.50 7.29 58 < .0001 0.96

City Neutral-negative 0.08 -0.08 0.23 0.99 57 0.329 0.13

**Positive-negative 0.46 0.31 0.61 6.12 57 < .0001 0.80**

**Positive-neutral 0.38 0.27 0.49 6.99 57 < .0001 0.92**

**Pearson Neutral-negative -0.34 -0.57 -0.11 3.02 57 0.004 0.40**

Positive-negative 0.11 -0.13 0.34 0.90 57 0.372 0.12

**Positive-neutral 0.45 0.27 0.62 5.09 57 < .0001 0.67**

*Table S3 (continued)*

**Confidence interval limits**

**Predictor Scale Level Comparison Difference lower upper *t* df *p* Cohen’s *d***

**Affective Overall Neutral-negative 0.57 0.49 0.65 14.04 58 < .0001 1.83**

**response Positive-negative 1.25 1.13 1.37 20.34 58 < .0001 2.65**

**Positive-neutral 0.68 0.60 0.76 17.15 58 < .0001 2.23**

**Strength Overall Neutral-negative -0.55 -0.79 -0.30 4.49 57 < .0001 0.59**

**of affective response Positive-negative 0.25 0.01 0.49 2.11 58 0.039 0.27**

**Positive-neutral 0.81 0.55 1.06 6.25 58 < .0001 0.81**

**Group Expression Overall Neutral-negative 0.85 0.81 0.9 40.72 70 < .0001 4.83**

**Positive-negative 1.88 1.85 1.9 136 70 < .0001 16.14**

**Positive-neutral 1.02 0.98 1.06 50.17 70 < .0001 5.95**

**Intensity Overall Neutral-negative -0.8 -0.96 -0.63 9.76 70 < .0001 1.16**

Positive-negative -0.09 -0.21 0.03 1.43 70 0.157 0.17

**Positive-neutral 0.71 0.55 0.87 9 70 < .0001 1.017**

**Midwives Neutral-negative -0.92 -1.11 -0.72 9.51 40 < .0001 1.49**

**Positive-negative -0.18 -0.3 -0.06 3.08 40 0.004 0.48**

**Positive-neutral 0.73 0.54 0.93 7.5 40 < .0001 1.17**

*Table S2 (continued)*

**Confidence interval limits**

**Predictor Scale Level Comparison Difference lower upper *t* df *p* Cohen’s *d***

**Neon. nurses Neutral-negative -0.59 -1 -0.18 3.14 11 0.009 0.91**

**Positive-negative 0.5 0.14 0.87 3.03 11 0.011 0.88**

**Positive-neutral 1.09 0.65 1.54 5.4 11 0.0002 1.56**

**Gen. publ. Neutral-negative -0.66 -1.08 -0.25 3.37 17 0.004 0.79**

**Positive-negative -0.26 -0.49 -0.04 2.45 17 0.025 0.58**

**Positive-neutral 0.4 0.1 0.71 2.79 17 0.013 0.66**

**Clarity Overall Neutral-negative -0.77 -0.95 -0.59 8.59 70 < .0001 1.02**

Positive-negative 0.11 -0.01 0.23 1.87 70 0.066 0.22

**Positive-neutral 0.88 0.72 1.05 10.53 70 < .0001 1.25**

**Midwives Neutral-negative -0.98 -1.22 -0.73 8.03 40 < .0001 1.25**

Positive-negative 0.06 -0.08 0.21 0.91 40 0.367 0.14

**Positive-neutral 1.04 0.82 1.26 9.48 40 < .0001 1.48**

**Neon. nurses Neutral-negative -0.51 -0.92 -0.1 2.71 11 0.02 0.78**

**Positive-negative 0.58 0.24 0.92 3.79 11 0.003 1.09**

**Positive-neutral 1.09 0.63 1.56 5.17 11 0.0003 1.49**

**Gen. publ. Neutral-negative -0.48 -0.81 -0.16 3.13 17 0.006 0.74**

Positive-negative -0.1 -0.29 0.09 1.08 17 0.296 0.25

**Positive-neutral 0.38 0.16 0.6 3.7 17 0.002 0.87**

*Table S3 (continued)*

**Confidence interval limits**

**Predictor Scale Level Comparison Differencelower upper *t* df *p* Cohen’s *d***

**Genuineness Overall** Neutral-negative 0.07 -0.08 0.22 0.89 70 0.375 0.11

**Positive-negative 0.54 0.4 0.68 7.8 70 < .0001 0.93**

**Positive-neutral 0.47 0.35 0.6 7.51 70 < .0001 0.89**

**Midwives** Neutral-negative 0.09 -0.11 0.3 0.91 40 0.368 0.14

**Positive-negative 0.56 0.36 0.75 5.84 40 < .0001 0.91**

**Positive-neutral 0.46 0.33 0.6 6.86 40 < .0001 1.07**

**Neon. nurses** Neutral-negative 0.03 -0.56 0.63 0.12 11 0.91 0.03

**Positive-negative 0.97 0.64 1.29 6.58 11 < .0001 1.9**

**Positive-neutral 0.93 0.43 1.43 4.11 11 0.002 1.19**

**Gen. publ.** Neutral-negative 0.04 -0.16 0.24 0.41 17 0.685 0.1

**Positive-negative 0.23 0.04 0.42 2.58 17 0.02 0.61**

**Positive-neutral 0.19 0.04 0.34 2.71 17 0.015 0.64**

**Affective Overall Neutral-negative 0.62 0.54 0.7 15.43 70 < .0001 1.83**

**response Positive-negative 1.32 1.2 1.43 22.94 70 < .0001 2.72**

**Positive-neutral 0.7 0.62 0.77 19.34 70 < .0001 2.29**

**Midwives Neutral-negative 0.58 0.47 0.69 10.75 40 < .0001 1.68**

**Positive-negative 1.28 1.14 1.41 19 40 < .0001 2.97**

**Positive-neutral 0.7 0.61 0.8 14.9 40 < .0001 2.33**

*Table S3 (continued)*

**Confidence interval limits**

**Predictor Scale Level Comparison Difference lower upper *t* df *p* Cohen’s *d***

**Neon. nurses Neutral-negative 0.89 0.69 1.09 9.76 11 < .0001 2.82**

**Positive-negative 1.66 1.47 1.86 18.5 11 < .0001 5.34**

**Positive-neutral 0.78 0.65 0.91 13.56 11 < .0001 3.91**

**Gen. publ. Neutral-negative 0.55 0.42 0.69 8.52 17 < .0001 2.01**

**Positive-negative 1.18 0.88 1.48 8.37 17 < .0001 1.97**

**Positive-neutral 0.63 0.45 0.8 7.41 17 < .0001 1.75**

**Strength Overall Neutral-negative -0.43 -0.65 -0.21 3.91 61 0.0002 0.5**

**of affective response Positive-negative 0.35 0.22 0.48 5.48 61 < .0001 0.7**

**Positive-neutral 0.78 0.56 1 7.08 61 < .0001 0.9**

*Table S4: Summary of the pairwise differences from Table S2. N, 0, and P stand for the negative, the neutral and the positive category, respectively. For example, P=N>0 signifies that positive and negative image received a higher rating than neutral images, but that the rating for positive and negative images did not differ significantly. Results for subsets (e.g., black/white images) are reported only if the corresponding predictor (e.g., color) interacted with the factor image category.*

**Analysis by**

**Color Infant gender Rater gender**

**Overall Black/white Color Overall Female Male Overall Female Male**

**Expression** P>0>N P>0>N P>0>N P>0>N −− −− P>0>N −− −−

**Intensity** N>P>0 −− −− P=N>0 −− −− P=N>0 P=N>0 ns

**Clarity** P=N>0 −− −− P=N>0 −− −− P=N>0 P>N>0 ns

**Genuineness** P>0=N −− −− P>0=N −− −− P>0=N −− −−

**Affect. resp.** P>0>N −− −− P>0>N −− −− P>0>N −− −−

**Strength of**

**affect. resp.** P=N>0 −− −− P>0>N −− −− P>N>0 −− −−

**Infant age** **Source**  **Group**

**Overall Young Old Overall City Pearson Overall Midwives Neon. nurses Gen. publ.**

**Expression** P>0>N −− −− P>0>N −− −− P>0>N −− −− −−

**Intensity** P=N>0 −− −− N>P>0 N>P>0 N>P>0 P=N>0 N>P>0 P>N>0 N>P>0

**Clarity** P=N>0 −− −− P=N>0 P=N>0 N>P>0 P=N>0 P=N>0 P>N>0 P=N>0

**Genuineness** P>0=N −− −− P>0=N P>0=N P=N>0 P>0=N P>0=N P>0=N P>0=N

**Affect. resp.** P>0>N P>0>N P>0>N P>0>N −− −− P>0>N P>0>N P>0>N P>0>N

**Strength of**

**affect. resp.** P>N>0 −− −− P>N>0 −− −− P>N>0 −− −− −−

S.2.2 DV: Clarity

For clarity, we observed a main effect of color, *F*(1,57) = 8.26, *p* = 0.007, η^2^_p_ = 0.127, suggesting that the clarity ratings were higher for black/white images (*M* = 3.47, *SD* = .69) than for color images (*M* = 3.33, *SD* = 0.87). A main effect of category was also found, showing that neutral images were rated as less clear than positive or negative images (see Table S1).

Except for main effects of category, no main effects or interactions were observed for intensity, genuineness, internal emotion and strength of internal emotion.

*Table S5. Results of ANOVAs assessing the influence of the factor Color (i.e., color vs. black/white images) on the different rating scales. Results are shown only for rating scales that showed significant effects.*

| **Scale** | **#excl.** | **Predictor** | **df’s** | **F** | **P** | **η^2^_p_** |
| --- | --- | --- | --- | --- | --- | --- |
| Expression | 0 | Image category | 2, 114 | 4260 | <.0001 | .897 |
|  |  | Color | 1, 57 | .167 | .68 | .003 |
|  |  | Interaction | 2, 114 | 4.55 | .013 | .074 |
| Clarity | 0 | Image category | 2, 114 | 60.24 | <.0001 | 0.514 |
|  |  | Color | 1, 57 | 8.258 | .005 | 0.127 |
|  |  | Interaction | 2, 114 | 0.4894 | .614 | 0.0085 |

*Note.* Only significant results are reported here. F values have been rounded to 2 decimal places.

Table S6*. Results of follow-up ANOVAs assessing the influence of the factor Color (i.e., color vs. black/white images) on the different rating scales.*

| **Scale** | **#excl.** | **Image subset** | **Df’s** | **F** | **p** | **η^2^_p_** |
| --- | --- | --- | --- | --- | --- | --- |
| Expression | 0 | Black/white | 2,114 | 4109 | <.0001 | .988 |
|  |  | Color | 2,114 | 1855 | <.0001 | .97 |
| Clarity | 0 | Black/white | 2, 114 | 64.19 | <.0001 | 0.53 |
|  |  | Color | 2,114 | 32.43 | <.0001 | 0.363 |

*Note.* F values have been rounded to 2 decimal places.

*Table S7. Follow up descriptive statistics assessing the influence of the factor Color (i.e., color vs. black/white images) on the different rating scales.*

|  |  | **Black/white** | | **Color** | |
| --- | --- | --- | --- | --- | --- |
|  |  | *M* | *SD* | *M* | *SD* |
| Expression | Negative | 1.06734743 | 0.08646731 | 1.0402299 | 0.1475966 |
|  | Neutral | 1.90237795 | 0.15564425 | 1.9525862 | 0.1847777 |
|  | Positive | 2.93667115 | 0.07152127 | 2.8947007 | 0.1721384 |
| Clarity | Negative | 3.7491019 | 0.4811382 | 3.5827586 | 0.9062086 |
|  | Neutral | 2.9108425 | 0.7350093 | 2.7278736 | 0.7539428 |
|  | Positive | 3.7570881 | 0.4286160 | 3.6860352 | 0.5879939 |

*Note.* For expression 1 = negative, 2 = neutral, 3 = positive. For clarity 1 = not clear; 5 = very clear

**S3 Male vs. Female Raters**

We analyzed the different rating scales in two-way ANOVAs with the within-subject factor expression category (negative vs. positive vs. neutral) and the between-subjects factor gender of the rater. All analyses detected the expected main effects of image category (*p* < .001). As shown in Tables S3 and S4, these main effects showed that (i) positive images were rated as more positive than neutral images, which were rated as more positive than negative images; that (ii) positive and negative images were rated as more intense than neutral images, with no difference between positive and negative images; that (iii) positive and negative images were rated as clearer than neutral images, with no difference between positive and negative images; that (iv) positive images were rated as more genuine than neutral and negative images, with no difference between neutral and negative images; that (v) the affective response reflected the valence of the image; and that (vi) positive image elicited a stronger affective response than negative images, which elicited a stronger response than neutral images in turn. Below, we will thus focus on the main effect of gender of the rater and the interaction.

S3.1 DV: Intensity

The analysis of the intensity rating revealed a significant interaction between the category of the image and the gender of the rater of the images *F*(2,132) = 3.44, *p* = 0.04, η^2^_p_ = 0.0245. Follow-up ANOVAS suggested that the effect of image category was stronger for female raters, *F*(2,122) = 73.83, *p* < .0001, η^2^_p_ = .548, than for male raters, *F*(2,10) = 0.55, *p* = 0.593, η^2^_p_ = 0.099, though it should be noted that the proportion of male raters was extremely low. In female raters, the effect of image category reflected that they rated positive and negative images as more intense than neutral images, with no difference between positive and negative images (see Tables S3 and S4). In male raters, these effects did not reach significance.

S3.2 DV: Clarity

Similar results were obtained in the analysis of the clarity rating, where we observed an interaction between image category and rater gender, *F*(2,132) = 5.2, *p* = 0.007, η^2^_p_ = 0.035. Again, follow-up analyses showed that the effect of image category was stronger for female raters, *F*(2,122) = 81.51, *p* < .0001, η^2^_p_ = .572, than for male raters, *F*(2,10) = 0.22, *p* = 0.806, η^2^_p_ = 0.04. As shown in Tables S3 and S4, female raters considered positive images as clearer than negative ones, which were rated as clearer than neutral ones, while these differences did not reach significance for male raters.

Table S8

*Results of ANOVAs assessing the influence of the factor Gender of rater (i.e., male vs. female raters) on the different rating scales. Results are shown only for rating scales that showed significant effects of Rater Gender or an interaction with this predictor.*

| **Scale** | **#excl.** | **Predictor** | **df’s** | **F** | **P** | **η^2^_p_** |
| --- | --- | --- | --- | --- | --- | --- |
| **Males vs. Female Raters** | | | | | | |
| Intensity | 0 | Image category | 1, 66 | 0.96 | .33 | 0.014 |
|  |  | Gender of Rater | 2, 132 | 69.86 | <.0001 | 0.502 |
|  |  | Interaction | 2, 132 | 3.44 | .035 | 0.025 |
| Clarity | 0 | Image category | 1, 66 | 0.02 | .877 | <.001 |
|  |  | Gender of Rater | 2, 132 | 77.21 | <.0001 | 0.52 |
|  |  | Interaction | 2, 132 | 5.20 | .007 | 0.035 |

*Note.* Only significant results are shown here. F values have been rounded to 2 decimal places

Table S9

*Follow up descriptive statistics assessing the influence of the factor Gender of rater (i.e., male vs. female rater) on the different rating scales.*

|  |  | **Female** | | **Male** | |
| --- | --- | --- | --- | --- | --- |
|  |  | **M** | **SD** | **M** | **SD** |
| Intensity | Negative | 3.66 | .48 | 3.35 | .56 |
|  | Neutral | 2.85 | .59 | 3.07 | .82 |
|  | Positive | 3.61 | .41 | 3.22 | .64 |
| Clarity | Negative | 3.62 | .62 | 3.68 | .58 |
|  | Neutral | 2.84 | .77 | 2.91 | .78 |
|  | Positive | 3.78 | .52 | 3.76 | .49 |

*Note.* 1 = not intense/clear; 5 = very intense/clear.

**S4 Male vs. Female Infants**

We analyzed the different rating scales in two-way within-subjects ANOVAs with the within-subject factors expression category (negative vs. positive vs. neutral) and gender of the infant. All analyses detected the expected main effects of image category (*p* < .001). As shown in Tables S3 and S4, these main effects showed that (i) positive images were rated as more positive than neutral images, which were rated as more positive than negative images; that (ii) positive and negative images were rated as more intense than neutral images, with no difference between positive and negative images; that (iii) positive and negative images were rated as clearer than neutral images, with no difference between positive and negative images; that (iv) positive images were rated as more genuine than neutral and negative images, with no difference between neutral and negative images; that (v) the affective response reflected the valence of the image; and that (vi) positive image elicited a stronger affective response than neutral images, which elicited a stronger response than negative images in turn. Below, we will focus on the main effects of infant gender and the interactions with this factor.

S4.1 DV: Intensity

There was a significant effect of gender on the intensity rating of the images *F*(1,70) = 5.55, *p* = 0.02, η^2^_p_  = 0.0735, showing that female infants received significantly higher ratings (*M* = 3.41, *SD* = .70) than male infants (*M* = 3.33, *SD* = .69).

DV: Strength of affective response

The gender of the infant also affected the strength of the internal emotion felt when looking at the infant, *F*(1,44) = 4.07, *p* = 0.05, η^2^_p_ = 0.0847, reflecting that female infants received higher ratings (*M* = 2.86, *SD* = .94) than male infants (*M* = 2.73, *SD* = .89).

*Table S10. Results of ANOVAs assessing the influence of the factor Gender of infant (i.e., male vs. female infants) on the different rating scales. Results are shown only for rating scales that showed significant effects of infant gender or interactions with this factor.*

| **Scale** | **#excl.** | **Predictor** | **df’s** | **F** | **P** | **η^2^_p_** |
| --- | --- | --- | --- | --- | --- | --- |
| **Males vs. Female Infants** | | | | | | |
| Intensity | 0 | Image category | 2, 140 | 69.09 | <.0001 | .497 |
|  |  | Gender of Infant | 1, 70 | 5.55 | <.021 | .007 |
|  |  | Interaction | 2, 140 | 0.46 | .634 | .006 |
| Strength | 26 | Image category | 2, 88 | 30.16 | <.0001 | .407 |
|  |  | Gender of Infant | 1, 44 | 4.07 | .050 | .085 |
|  |  | Interaction | 2, 88 | 1.38 | .256 | .031 |

*Note.* Only significant results are shown here. F values have been rounded to 2 decimal places.

**S5 Younger vs. Older Infants**

We analyzed the different rating scales in two-way within-subjects ANOVAs with the within-subject factors expression category (negative vs. positive vs. neutral) and age of the infant (up to 6 months vs. above). All analyses detected the expected main effects of image category (*p* < .001). As shown in Tables S3 and S4, these main effects showed that (i) positive images were rated as more positive than neutral images, which were rated as more positive than negative images; that (ii) positive and negative images were rated as more intense than neutral images, with no difference between positive and negative images; that (iii) positive and negative images were rated as clearer than neutral images, with no difference between positive and negative images; that (iv) positive images were rated as more genuine than neutral and negative images, with no difference between neutral and negative images; that (v) the affective response reflected the valence of the image; and that (vi) positive image elicited a stronger affective response than negative images, which elicited a stronger response than neutral images in turn. Below, we will focus on the main effect of infant age and the interactions.

S5.1 DV: Genuineness

The analysis of the genuineness rating yielded a significant main effect of infant age, *F*(1,70) = 9.87, *p* = .002, η^2^_p_ = 0.124, suggesting that younger infants were rated as being more genuine (*M* = 3.77, *SD* = 0.71) compared to older infants (*M* = 3.67, *SD* = 0.72).

S5.2 DV: Affective response

When affective response was used as the dependent variable (where one participant had to be excluded from analysis for not completing all cells of the design), a significant interaction between the category of the image and the age of the infant was found, *F*(2,138) = 4.21, *p* = 0.017, η^2^_p_ = 0.057. Follow up ANOVAs showed that the effect of image category was stronger for younger infants, *F*(2,138) = 417.8, *p* < .0001, η^2^_p_ = 0.858, than for older ones, *F*(2,138) = 345.9, *p* < .0001, η^2^_p_ = 0.834. However, as shown in Tables S3 and S4, all pairwise differences between image categories were significant for both age classes, though younger infants elicited affective responses closer to those intended (i.e. negative emotions for negative images) for all image categories, compared to older infants.

*Table S11. Results of ANOVAs assessing the influence of the factor age of infant (i.e., younger vs. older infants) on the different rating scales. Results are shown only for rating scales that showed significant main effects of or interactions with infant age.*

| **Scale** | **#excl.** | | **Predictor** | **df’s** | **F** | **P** | **η^2^_p_** |
| --- | --- | --- | --- | --- | --- | --- | --- |
| **Younger vs. Older Infants** | | | | | | | |
| Genuineness | 0 | | Image category | 2, 140 | 35.04 | <.0001 | .334 |
|  |  | | Age of Infant | 1, 70 | 9.87 | .002 | .124 |
|  |  | | Interaction | 2, 140 | 2.67 | .072 | .037 |
| Affective response | | 1 | Image category | 2, 138 | 451.70 | <.0001 | .867 |
|  | |  | Age of Infant | 1, 69 | 0.02 | .897 | .897 |
|  | |  | Interaction | 2, 138 | 4.21 | .017 | .057 |

*Note.* Only significant results are shown here. F values have been rounded to 2 decimal places.

Table S12

*Follow up descriptive statistics assessing the influence of the factor age of infant (i.e., younger vs. older infant) on the different rating scales.*

|  |  | Younger | | Older | |
| --- | --- | --- | --- | --- | --- |
|  |  | M | SD | M | SD |
| Genuineness | Negative | 3.61 | .72 | 3.41 | .78 |
|  | Neutral | 3.60 | .75 | 3.57 | .69 |
|  | Positive | 4.09 | .53 | 4.02 | .54 |
| Affective Response | Negative | 1.40 | .32 | 1.44 | .35 |
|  | Neutral | 2.04 | .29 | 2.07 | .23 |
|  | Positive | 2.79 | .23 | 2.73 | .26 |

*Note.* For Affective Response: 1 = negative, 2 = neutral, 3 = positive.

**S6. City vs. Pearson Images**

To assess criterion validity, we analyzed the different rating scales in two-way within-subjects ANOVAs with the within-subject factors expression category (negative vs. positive vs. neutral) and source of the image (Pearson database vs. City database). All of the Pearson’s images were included in this analysis, although 7 of them did not yield agreements of 75% (see below). The midwives and the general public completed these ratings. All analyses detected the expected main effects of image category (*p* < .001). As shown in Tables S3 and S4, these main effects showed that (i) positive images were rated as more positive than neutral images, which were rated as more positive than negative images; that (ii) positive were rated as more intense than negative images, which were rated as more intense than neutral images in turn; that (iii) positive and negative images were rated as clearer than neutral images, with no difference between positive and negative images; that (iv) positive images were rated as more genuine than neutral and negative images, with no difference between neutral and negative images; that (v) the affective response reflected the valence of the image; and that (vi) positive image elicited a stronger affective response than negative images, which elicited a stronger response than neutral images in turn. Below, we will thus focus on the main effects of source and its interactions.

Table S13.

*Pearson’s images with low agreement ratings*

|  | imageID | Agreement Rating |
| --- | --- | --- |
| Negative | 139 | 0.5666667 |
| Neutral | 118 | 0.6206897 |
| Neutral | 121 | 0.4047619 |
| Neutral | 136 | 0.6206897 |
| Neutral | 137 | 0.6551724 |
| Neutral | 138 | 0.6666667 |
| Positive | 134 | 0.7419355 |

S6.1 DV: Expression

In the analysis of the expression rating, we observed a significant effect of source, *F*(1,58) = 13.33, *p* < 0.001, η^2^_p_ = 0.187, suggesting that the ratings were somewhat higher (i.e., more positive) for the City database (*M* = 1.97, *SD* = 0.77) compared to the Pearson database (*M* = 1.91 SD = 0.77).

S6.2 DV: Intensity

When intensity was used as the dependent variable, there was a significant effect of source, *F*(1,58) = 4.16, *p* = 0.046, η^2^_p_ = 0.0669, showing that the Pearson images (*M* = 3.47, *SD* = 0.83) were rated as more intense than the City images (*M* = 3.40, *SD* = 0.62). There was also a significant interaction between source and image category, *F*(2,116) = 18.94, *p* < .0001, η^2^_p_ = 0.246. Follow-up ANOVAs revealed that the effect of image category was somewhat stronger for the Pearson database, *F*(2,116) = 77.73, *p* < .0001, η^2^_p_ = 0.573, than for the City Database, *F*(2,116) = 64.76, *p* < .0001, η^2^_p_ = 0.528. As shown in Tables S3 and S4, participants rated negative images as more intense than positive images, which were rated as more intense than neutral images. However, this is effect was somewhat more pronounced for the Pearson database, especially for negative images. Descriptive statistics show that this interaction is likely to be driven by the negative images which were rated as more intense in the Pearson image set than the City database. City’s positive images were rated as somewhat more intense.

S6.2 DV: Clarity

The analysis of the clarity rating revealed a significant interaction between image category and source, *F*(2,116) = 25.44, *p* < .0001, η^2^_p_ = 0.305. Follow-up ANOVAs indicated that the effect of image category was stronger for the Pearson database, *F*(2,116) = 72.35, *p* < .0001, η^2^_p_ = 0.555, than for the City Database, *F*(2,116) = 64.2, *p* < .0001, η^2^_p_ = 0.525. As shown in Tables S3 and S4, in Pearson’s image set, the negative images were rated as the clearest, followed by the positive images, which were rated clearer than neutral images. For the City database, in contrast, negative and positive images were rated clearer than neutral images, with no difference between negative and positive images.

S6.3 DV: Genuineness

For the analysis of the genuineness rating, one participant needed to be excluded. The analysis yielded a main effect of category, with the positive images being rated as more genuine than neutral or negative images, with no difference between neutral and negative images, *F*(2,114) = 14.44, *p* < .0001, η^2^_p_ = 0.202 (see Tables S3 and S4). There was also an interaction between image category and source, *F*(2,114) = 12.83, *p* < .00001, η^2^_p_ = .184, suggesting that the effect of image category was stronger for the City database, *F*(2,114) = 24.85, *p* < .0001, η^2^_p_ = 0.304, than for the Pearson database, *F*(2,114) = 9.49, *p* = .0002, η^2^_p_ = .143. As shown in Tables S3 and S4, positive images were rated as more genuine than either neutral or negative images in the City database, with no difference between neutral and negative images. In contrast, in the Pearson database, neutral images were rated as less genuine than positive or negative images, with no difference between positive and negative images.

S6.4 DV: Affective Response

For the analysis of the rating of affective response, one participant needed to be excluded. The analysis revealed a significant effect of source, *F*(1,57) = 17.19, *p* < .001, η^2^_p_ = .232, suggesting that the ratings were higher for the City database (*M* = 2.06, *SD* = .58) than for the Pearson database (*M* = 1.98, *SD* = .61).

Table S14

*Results of ANOVAs assessing the influence of the factor Source (i.e., City Database vs. Pearson Image Set) on the different rating scales. Results are shown only for rating scales that showed significant main effects of or interactions with the predictor source.*

| **Scale** | **#excl.** | **Predictor** | **df’s** | | **F** | **P** | **η^2^_p_** |
| --- | --- | --- | --- | --- | --- | --- | --- |
| **Pearson vs. City Images** | | | | | | | |
| Expression | 0 | Image category | | 2, 116 | 2694 | <.0001 | .979 |
|  |  | Source of Image | | 1, 58 | 13.33 | <.0001 | .187 |
|  |  | Interaction | | 2, 116 | 1.62 | .2019 | .027 |
| Intensity | 0 | Image category | | 2, 116 | 97.20 | <.0001 | .627 |
|  |  | Source of Image | | 1, 58 | 4.16 | .046 | .066 |
|  |  | Interaction | | 2, 116 | 18.94 | <.0001 | .246 |
| Clarity | 0 | Image category | | 2, 116 | 84.62 | <.0001 | 0.593 |
|  |  | Source | | 1, 58 | 0.01 | 0.929 | 0.000 |
|  |  | Interaction | | 2, 116 | 25.44 | <.0001 | 0.305 |
| Genuineness | 1 | Image category | | 2, 114 | 14.44 | <.0001 | 0.202 |
|  |  | Source | | 1, 57 | 0.04 | 0.840 | .001 |
|  |  | Interaction | | 2,114 | 12.83 | <.0001 | 0.184 |
| Affective Response | 26 | Image category | | 2, 114 | 340.60 | <.0001 | .857 |
|  |  | Source | | 1,57 | 17.19 | .0001 | .232 |
|  |  | 2,114 | | .180 | .84 | .003 |  |

*Note.* Only significant results are reported here. F values have been rounded to 2 decimal places.

Table S15

*Follow up descriptive statistics assessing the influence of the factor Source (i.e., City Database vs. Pearson Image Set) on the different rating scales*

|  |  | **City** | | **Pearson** | |
| --- | --- | --- | --- | --- | --- |
|  |  | **M** | **SD** | **M** | **SD** |
| Expression | Negative | 1.06620595 | 0.08616592 | 1.0537225 | 0.1049083 |
|  | Neutral | 1.90403256 | 0.15481920 | 1.8276836 | 0.3163884 |
|  | Positive | 2.93532321 | 0.07165400 | 2.8489642 | 0.2406411 |
| Intensity | Negative | 3.7445693 | 0.4057327 | 4.1755631 | 0.5978158 |
|  | Neutral | 2.9058225 | 0.6496706 | 2.8710923 | 0.7263951 |
|  | Positive | 3.5389688 | 0.4298563 | 3.3701507 | 0.5752467 |
| Clarity | Negative | 3.7420550 | 0.4800339 | 4.1527331 | 0.7233263 |
|  | Neutral | 2.9165909 | 0.7299821 | 2.7712806 | 0.8112404 |
|  | Positive | 3.7563626 | 0.4249415 | 3.4809793 | 0.5876661 |
| Genuineness | Negative | 3.6560450 | 0.6398594 | 3.9200042 | 0.8990934 |
|  | Neutral | 3.7321501 | 0.5515531 | 3.5797893 | 0.8990934 |
|  | Positive | 4.1145368 | 0.4704406 | 4.0267241 | 0.5823346 |
| Affective Response | Negative | 1.4482222 | 0.3055086 | 1.3823120 | 0.3382781 |
|  | Neutral | 2.0279866 | 0.2276781 | 1.9315134 | 0.2899560 |
|  | Positive | 2.7178908 | 0.2210042 | 2.6363027 | 0.3331940 |

*Note.* For expression & affective response 1 = negative, 2 = neutral, 3 = positive. For clarity/intensity/genuineness 1 = not clear/intense/genuine 5 = very clear/intense/genuine.

**S7 Difference between groups**

We compared the ratings across the three groups (midwives, neonatal nurses, general public) in all rating scales by performing ANOVAs with the within-participants factor image category and the between-participant factor group. A main effect of image category was found for all analyses. As shown in Tables S3 and S4, these main effects showed that (i) positive images were rated as more positive than neutral images, which were rated as more positive than negative images; that (ii) positive and negative images were rated as more intense than neutral images, with no difference between positive and negative images; that (iii) positive and negative images were rated as clearer than neutral images, with no difference between positive and negative images; that (iv) positive images were rated as more genuine than neutral and negative images, with no difference between neutral and negative images; that (v) the affective response reflected the valence of the image; and that (vi) positive image elicited a stronger affective response than negative images, which elicited a stronger response than neutral images in turn.

S7.1 DV: Expression

The analysis of the expression ratings yielded a main effect of group, *F*(2,68) = 3.59, *p* = 0.03, η^2^_p_ = 0.0954. Post-hoc test (Tukey’s HSD’s) revealed that the neonatal nurses had higher ratings compared midwives and compared to the general public (*p*’s < .0002), while midwives and the general public did not differ significantly.

S7.2 DV: Intensity

The analyses of the intensity rating revealed a significant interaction between group and image category, *F*(4,136) = 4.72, *p* = .001, η^2^_p_ = 0.0612. Follow-up ANOVAs revealed that the effect of image category was more pronounced for neonatal nurses, *F*(2,80) = 63.25, *p* < .00001, η^2^_p_ = 0.613, than for midwives, *F*(2,22) = 17.32, *p* < .0001, η^2^_p_ = 0.612 and for the general public, *F*(2,34) = 9.44, *p* = 0.0005, η^2^_p_ = .357. As shown in Tables S3 and S4, the midwives and the neonatal nurses rated the positive and negative images as the most intense ones. For the general public, the negative images were rated as the most intense.

S7.3 DV: Clarity

The analyses of the clarity rating revealed a significant interaction between group and image category, *F*(4,136) = 5.89, *p* = 0.0002, η^2^_p_ = 0.071. Follow-up ANOVAs revealed that the effect of image category was more pronounced for neonatal nurses, *F*(2,80) = 64.26, *p* < .00001, η^2^_p_ = 0.616, than for midwives, *F*(2,22) = 17.31, *p* < .0001, η^2^_p_ = 0.611 and the general public, *F*(2,34) = 9.01, *p* = 0.0007, η^2^_p_ = .348. As shown in Tables S3 and S4, the midwives and the general public saw positive and negative images as clearer than neutral images, with no difference with the former two categories. In contrast, the neonatal nurses rated that positive images as clearer than the negative images, which they rated as clearer than the neutral images in turn.

S7.4 DV: Genuineness

When genuineness was used as the dependent variable, a main effect of group, *F*(2,68) = 11.85, *p* < .001, η^2^_p_ = 0.258, was found, showing that neonatal nurses rated all images as being less genuine. Post hoc tests (Tukey’s HSD) showed that the main effect reflects that the neonatal nurses (*M* = 3.15) gave lower ratings than either midwives (*M* = 3.76) or the general public (*M* = 3.99; *p*’s ≤ .002), while midwives and the general public did not differ statistically. An interaction between group and image category, *F*(4,136) = 4.13, *p* = 0.003, η^2^_p_ = 0.0719 was found. The interaction reflects that the effect of image category was more pronounced for midwives, *F*(2,80) = 22.21, *p* < .0001, η^2^_p_ = 0.357, than for neonatal nurses, *F*(2,22) = 12.34, *p* = 0.0003, η^2^_p_ = 0.529 or the general public, F(2,34) = 4.16, p = 0.024, η^2^_p_ = .197.

S7.5 DV: Affective Response

The analysis of the rating of affective response revealed an interaction between image category and group, *F*(4,136) = 3.69, *p* = 0.007, η^2^_p_ = 0.014. Follow-up ANOVAs suggest that the effect of image category is less pronounced for the general public, *F*(2,34) = 66.91, *p* < .0001, η^2^_p_ = 0.797, than for midwives, *F*(2,80) = 255.4, *p* < .00001, η^2^_p_ = 0.865 or neonatal nurses, *F*(2,22) = 212, *p* < .0001, η^2^_p_ = 0.951. As shown in Tables S3 and S4, while images elicited internal emotions expected by the image category (i.e. negative emotions for negative images), this relationship was the strongest for the neonatal nurses.

Table S16

*Results of ANOVAs assessing the influence of the factor Group (i.e., Midwives, vs. Neonatal Nurses, vs. General Public) on the different rating scales. Results are shown only for rating scales that showed significant main effects of or interactions with group*

| **Scale** | **#excl.** | **Predictor** | **Within?** | **df’s** | **F** | **P** | **η^2^_p_** |
| --- | --- | --- | --- | --- | --- | --- | --- |
|  | **Midwives vs. Neonatal Nurses vs. General Public** | | | | | | |
| Expression | 0 | Image category | No | 2, 68 | 3.59 | .033 | .095 |
|  |  | Group | No | 2, 136 | 5139 | <.0001 | .986 |
|  |  | Interaction | No | 4, 136 | 1.51 | .203 | .000 |
| Intensity | 0 | Image category | No | 2, 68 | 1.27 | .290 | .036 |
|  |  | Group | No | 2, 136 | 76.91 | <.0001 | .498 |
|  |  | Interaction | No | 4, 136 | 4.72 | .001 | .061 |
| Clarity | 0 | Image category | No | 2, 68 | 2.28 | .110 | .062 |
|  |  | Group | No | 2, 136 | 84.98 | <.0001 | .516 |
|  |  | Interaction | No | 4, 136 | 5.89 | .0002 | .071 |
| Genuineness | 0 | Image category | No | 2, 68 | 11.85 | <.0001 | .258 |
|  |  | Group | No | 2, 136 | 38.71 | <.0001 | .337 |
|  |  | Interaction | No | 4, 136 | 4.13 | <.003 | .072 |
| Affective Response | 0 | Image category | No | 2, 68 | 2.29 | .109 | .063 |
|  |  | Group | No | 2, 136 | 451.20 | <.0001 | .857 |
|  |  | Interaction | No | 4, 136 | 3.69 | .007 | .014 |
| Strength | 9 | Midwives | No | 2, 118 | 32.97 | <.0001 | .346 |
|  |  | Neonatal Nurses | No | 2, 59 | 3.05 | .05 | .094 |
|  |  | General | No | 4, 118 | 1.63 | .172 | .034 |

*Note.* Only significant results are reported here. F values have been rounded to 2 decimal places.

Table S17

*Follow up descriptive statistics assessing the influence of the factor Group (i.e., Midwives, vs. Neonatal Nurses, vs. General Public) on the different rating scales.*

|  |  | **Midwives** | | **Neonatal Nurses** | | | | **General public** | | | |
| --- | --- | --- | --- | --- | --- | --- | --- | --- | --- | --- | --- |
|  |  | **M** | **SD** | **M** | | **SD** | | **M** | | **SD** | |
| Expression | Negative | 1.08 | .10 | 1.08 | | .09 | | 1.04 | | .05 | |
|  | Neutral | 1.89 | .18 | 2.01 | | .18 | | 1.93 | | .09 | |
|  | Positive | 2.94 | .07 | 2.99 | | .03 | | 2.93 | | .08 | |
| Intensity | Negative | 3.78 | .34 | 3.23 | .73 | | 3.66 | | .53 | |  |
|  | Neutral | 2.87 | .60 | 2.64 | .68 | | 3.00 | | .77 | |  |
|  | Positive | 3.60 | .37 | 3.73 | .56 | | 3.40 | | .53 | |  |
| Clarity | Negative | 3.75 | .42 | 3.16 | .73 | | 3.73 | | .60 | |  |
|  | Neutral | 2.77 | .73 | 2.65 | .79 | | 3.25 | | .64 | |  |
|  | Positive | 3.81 | .36 | 3.74 | .61 | | 3.63 | | .54 | |  |
| Genuineness | Negative | 3.55 | .60 | 2.82 | .57 | | 3.90 | | .67 | |  |
|  | Neutral | 3.64 | .53 | 2.85 | .87 | | 3.93 | | .55 | |  |
|  | Positive | 4.11 | .43 | 3.79 | .60 | | 4.13 | | .56 | |  |
| Affective Response | Negative | 1.46 | .29 | 1.29 | | .31 | | 1.46 | | .37 | |
|  | Neutral | 2.04 | .25 | 2.18 | | .19 | | 2.01 | | .17 | |
|  | Positive | 2.74 | .19 | 2.96 | | .04 | | 2.64 | | .32 | |

*Note.* For expression & affective response 1 = negative, 2 = neutral, 3 = positive. For clarity/intensity/genuineness 1 = not clear/intense/genuine 5 = very clear/intense/genuine.

S7.6 DV: Strength

When strength of affective response was used as the dependent variable, a marginal effect of group was found *F*(2,59) = 3.05, *p* = 0.055, η^2^_p_ = 0.0937 (9 participants excluded). Post-hoc tests (Tukey’s HSD) revealed that the ratings in the general public were marginally lower than in neonatal nurses and midwives.

Table S18

*Descriptive Statistics of the factor Group (i.e., Midwives, vs. Neonatal Nurses, vs. General Public) on the Strength Scale*

|  | **N** | **M** | **SD** | **SE** | **CI** |
| --- | --- | --- | --- | --- | --- |
| Midwives | 41 | 3.11 | 0.65 | 0.10 | 0.21 |
| Neonatal Nurses | 12 | 3.27 | 0.43 | 0.12 | 0.27 |
| General Public | 18 | 2.71 | 0.76 | 0.18 | 0.38 |

*Note.* 1 = weak, 5 = strong internal emotions.

Table S19

*Tukey’s HSD for factor Group (i.e., Midwives, vs. Neonatal Nurses, vs. General Public)*

|  |  | **All groups** | | | |
| --- | --- | --- | --- | --- | --- |
| **Scale** | **Condition** | **Difference** | **95% Confidence interval lower bound** | **95% Confidence interval upper bound** | **Adjusted P value** |
| Expression | Neonatal Nurses - Midwives | .113 | .053 | .174 | <.0001 |
|  | General public - Midwives | -.009 | -.062 | .043 | .904 |
|  | General Public – Neonatal Nurses | -.122 | -.019 | -.054 | .0002 |
| Genuineness | Neonatal Nurses - Midwives | .051 | -.908 | -.175 | .002 |
|  | General public - Midwives | .201 | -.115 | .517 | .285 |
|  | General Public – Neonatal Nurses | .742 | .326 | 1.158 | <.001 |
| Internal emotion | Neonatal Nurses - Midwives | .123 | .016 | .232 | .021 |
|  | General public - Midwives | -.047 | -.141 | .046 | .447 |
|  | General Public – Neonatal Nurses | -.012 | .294 | -.048 | .004 |
| Strength | Neonatal Nurses – Midwives | .157 | -.353 | .667 | .742 |
|  | General public – Midwives | -.402 | -.842 | .037 | .080 |
|  | General public – Neonatal nurses | -.560 | -1.138 | .020 | .061 |

*Note.* Numbers have been rounded to 3 decimal places.
